# 

**Published:** 1868-08

**Authors:** 


### CONTENTS.

ORIGINAL COMMUNICATIONS.

Address .......... 311
"Peace, Rude Boreas" .......... 319
Alveolar Abscess .......... 338
Os-Artificial .......... 346
A "Scientific" Estimate of Asphyxiation by Nitrous Oxide .......... 347

EDITORIAL.

The Law Regulating Dental Practice .......... 350
Indiana State Dental Society .......... 352
College Matters .......... 353
"Peace, Rude Boreas" .......... 354
Obituary .......... 354

